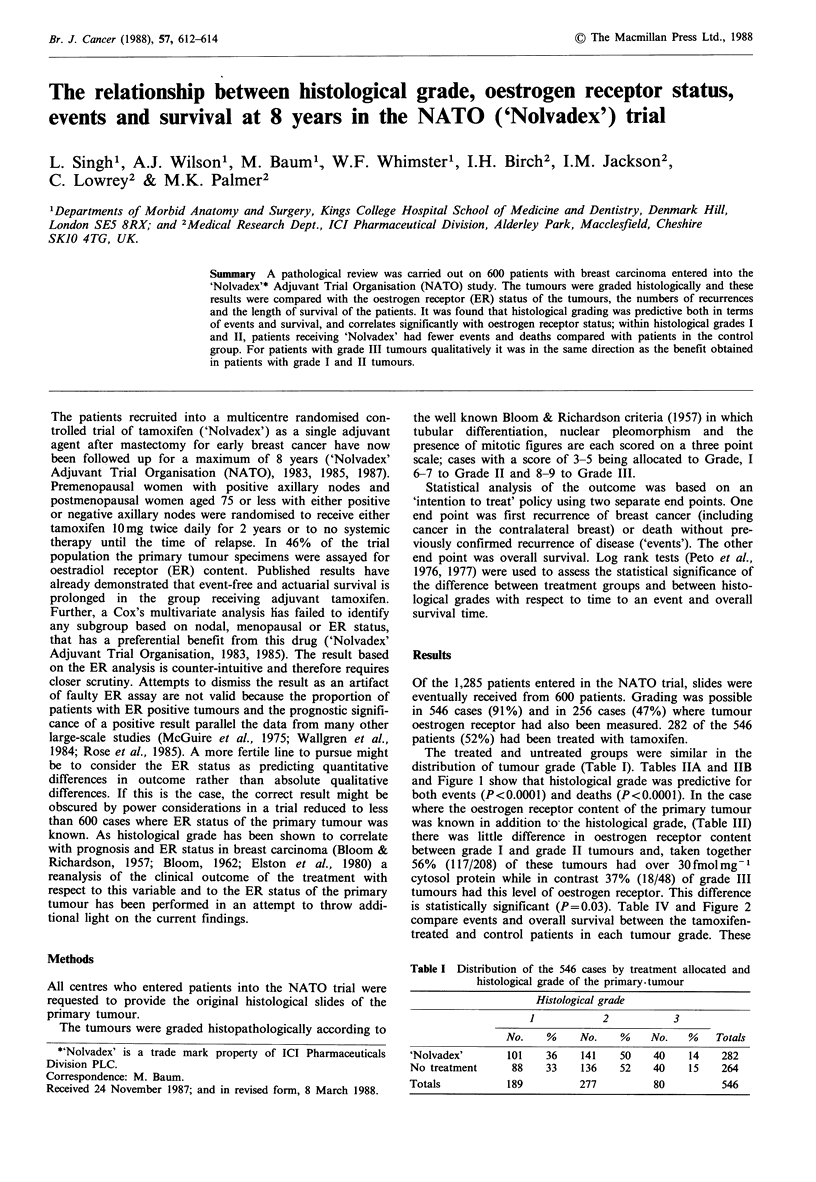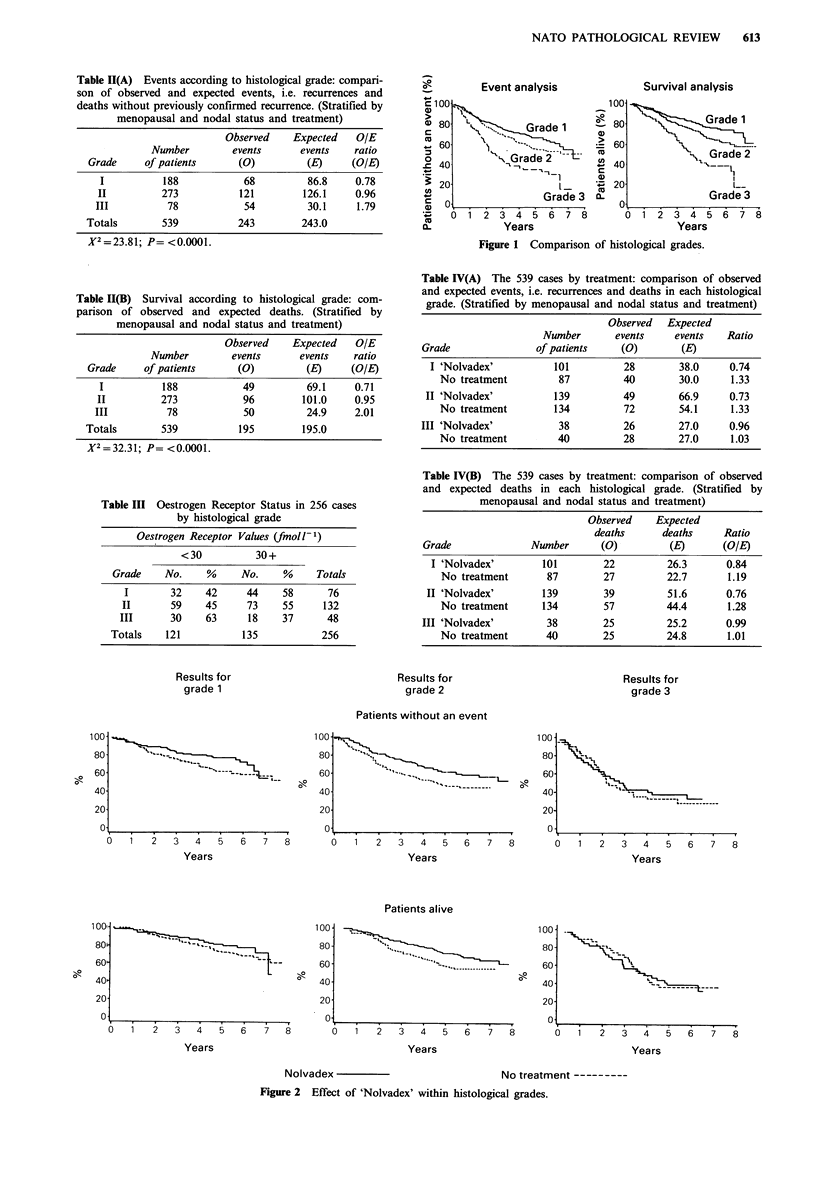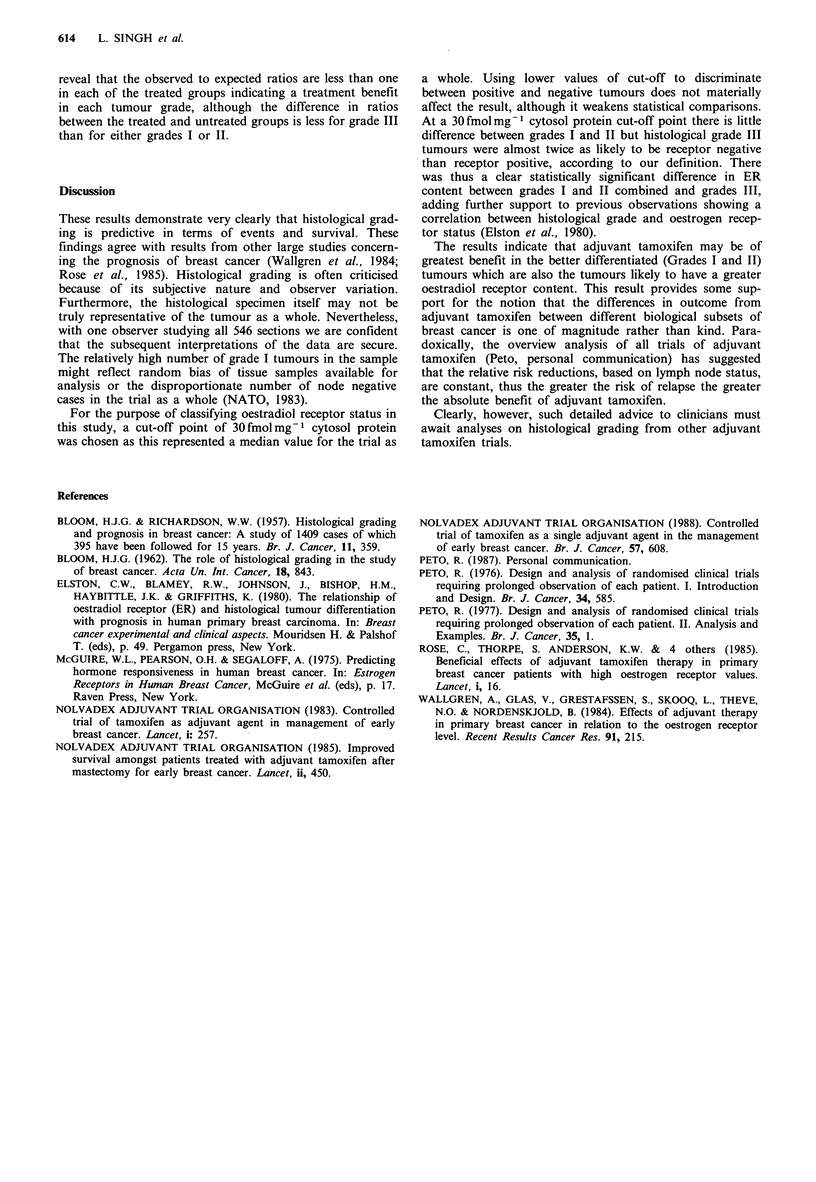# The relationship between histological grade, oestrogen receptor status, events and survival at 8 years in the NATO ('Nolvadex') trial.

**DOI:** 10.1038/bjc.1988.139

**Published:** 1988-06

**Authors:** L. Singh, A. J. Wilson, M. Baum, W. F. Whimster, I. H. Birch, I. M. Jackson, C. Lowrey, M. K. Palmer

**Affiliations:** Department of Morbid Anatomy, Kings College Hospital School of Medicine and Dentistry, Denmark Hill, London, UK.

## Abstract

A pathological review was carried out on 600 patients with breast carcinoma entered into the 'Nolvadex' Adjuvant Trial Organisation (NATO) study. The tumours were graded histologically and these results were compared with the oestrogen receptor (ER) status of the tumours, the numbers of recurrences and the length of survival of the patients. It was found that histological grading was predictive both in terms of events and survival, and correlates significantly with oestrogen receptor status; within histological grades I and II, patients receiving 'Nolvadex' had fewer events and deaths compared with patients in the control group. For patients with grade III tumours qualitatively it was in the same direction as the benefit obtained in patients with grade I and II tumours.


Br. J. Cancer (1988), 57, 612–614 © The Macmillan Press Ltd., 1988

### The relationship between histological grade, oestrogen receptor status, events and survival at 8 years in the NATO ('Nolvadex') trial

L. Singh[1], A.J. Wilson[1], M. Baum[1], W.F. Whimster[1], I.H. Birch[2], I.M. Jackson[2], C. Lowrey[2] & M.K. Palmer[2]

[1]*Departments of Morbid Anatomy and Surgery, Kings College Hospital School of Medicine and Dentistry, Denmark Hill, London SE5 8RX; and* [2]*Medical Research Dept., ICI Pharmaceutical Division, Alderley Park, Macclesfield, Cheshire SK10 4TG, UK.*

**Summary** A pathological review was carried out on 600 patients with breast carcinoma entered into the 'Nolvadex'* Adjuvant Trial Organisation (NATO) study. The tumours were graded histologically and these results were compared with the oestrogen receptor (ER) status of the tumours, the numbers of recurrences and the length of survival of the patients. It was found that histological grading was predictive both in terms of events and survival, and correlates significantly with oestrogen receptor status; within histological grades I and II, patients receiving 'Nolvadex' had fewer events and deaths compared with patients in the control group. For patients with grade III tumours qualitatively it was in the same direction as the benefit obtained in patients with grade I and II tumours.

The patients recruited into a multicentre randomised controlled trial of tamoxifen ('Nolvadex') as a single adjuvant agent after mastectomy for early breast cancer have now been followed up for a maximum of 8 years ('Nolvadex' Adjuvant Trial Organisation (NATO), 1983, 1985, 1987). Premenopausal women with positive axillary nodes and postmenopausal women aged 75 or less with either positive or negative axillary nodes were randomised to receive either tamoxifen 10 mg twice daily for 2 years or to no systemic therapy until the time of relapse. In 46% of the trial population the primary tumour specimens were assayed for oestradiol receptor (ER) content. Published results have already demonstrated that event-free and actuarial survival is prolonged in the group receiving adjuvant tamoxifen. Further, a Cox's multivariate analysis has failed to identify any subgroup based on nodal, menopausal or ER status, that has a preferential benefit from this drug ('Nolvadex' Adjuvant Trial Organisation, 1983, 1985). The result based on the ER analysis is counter-intuitive and therefore requires closer scrutiny. Attempts to dismiss the result as an artifact of faulty ER assay are not valid because the proportion of patients with ER positive tumours and the prognostic significance of a positive result parallel the data from many other large-scale studies (McGuire *et al.*, 1975; Wallgren *et al.*, 1984; Rose *et al.*, 1985). A more fertile line to pursue might be to consider the ER status as predicting quantitative differences in outcome rather than absolute qualitative differences. If this is the case, the correct result might be obscured by power considerations in a trial reduced to less than 600 cases where ER status of the primary tumour was known. As histological grade has been shown to correlate with prognosis and ER status in breast carcinoma (Bloom & Richardson, 1957; Bloom, 1962; Elston *et al.*, 1980) a reanalysis of the clinical outcome of the treatment with respect to this variable and to the ER status of the primary tumour has been performed in an attempt to throw additional light on the current findings.

### Methods

All centres who entered patients into the NATO trial were requested to provide the original histological slides of the primary tumour.

The tumours were graded histopathologically according to the well known Bloom & Richardson criteria (1957) in which tubular differentiation, nuclear pleomorphism and the presence of mitotic figures are each scored on a three point scale; cases with a score of 3–5 being allocated to Grade, I 6–7 to Grade II and 8–9 to Grade III.

Statistical analysis of the outcome was based on an 'intention to treat' policy using two separate end points. One end point was first recurrence of breast cancer (including cancer in the contralateral breast) or death without previously confirmed recurrence of disease ('events'). The other end point was overall survival. Log rank tests (Peto *et al.*, 1976, 1977) were used to assess the statistical significance of the difference between treatment groups and between histological grades with respect to time to an event and overall survival time.

### Results

Of the 1,285 patients entered in the NATO trial, slides were eventually received from 600 patients. Grading was possible in 546 cases (91%) and in 256 cases (47%) where tumour oestrogen receptor had also been measured. 282 of the 546 patients (52%) had been treated with tamoxifen.

The treated and untreated groups were similar in the distribution of tumour grade (Table I). Tables IIA and IIB and Figure 1 show that histological grade was predictive for both events ($P<0.0001$) and deaths ($P<0.0001$). In the case where the oestrogen receptor content of the primary tumour was known in addition to the histological grade, (Table III) there was little difference in oestrogen receptor content between grade I and grade II tumours and, taken together 56% (117/208) of these tumours had over 30 fmol mg$^{-1}$ cytosol protein while in contrast 37% (18/48) of grade III tumours had this level of oestrogen receptor. This difference is statistically significant ($P=0.03$). Table IV and Figure 2 compare events and overall survival between the tamoxifen-treated and control patients in each tumour grade. These

**Table I** Distribution of the 546 cases by treatment allocated and histological grade of the primary tumour

| | *Histological grade* | | | | | | |
|---|---|---|---|---|---|---|---|
| | *1* | | *2* | | *3* | | |
| | *No.* | *%* | *No.* | *%* | *No.* | *%* | *Totals* |
| 'Nolvadex' | 101 | 36 | 141 | 50 | 40 | 14 | 282 |
| No treatment | 88 | 33 | 136 | 52 | 40 | 15 | 264 |
| Totals | 189 | | 277 | | 80 | | 546 |

*'Nolvadex' is a trade mark property of ICI Pharmaceuticals Division PLC.
Correspondence: M. Baum.
Received 24 November 1987; and in revised form, 8 March 1988.

**Table II(A)** Events according to histological grade: comparison of observed and expected events, i.e. recurrences and deaths without previously confirmed recurrence. (Stratified by menopausal and nodal status and treatment)

| *Grade* | *Number of patients* | *Observed events (O)* | *Expected events (E)* | *O/E ratio (O/E)* |
|---|---|---|---|---|
| I | 188 | 68 | 86.8 | 0.78 |
| II | 273 | 121 | 126.1 | 0.96 |
| III | 78 | 54 | 30.1 | 1.79 |
| Totals | 539 | 243 | 243.0 | |

$X^2 = 23.81$; $P = <0.0001$.

**Table II(B)** Survival according to histological grade: comparison of observed and expected deaths. (Stratified by menopausal and nodal status and treatment)

| *Grade* | *Number of patients* | *Observed events (O)* | *Expected events (E)* | *O/E ratio (O/E)* |
|---|---|---|---|---|
| I | 188 | 49 | 69.1 | 0.71 |
| II | 273 | 96 | 101.0 | 0.95 |
| III | 78 | 50 | 24.9 | 2.01 |
| Totals | 539 | 195 | 195.0 | |

$X^2 = 32.31$; $P = <0.0001$.

**Table III** Oestrogen Receptor Status in 256 cases by histological grade

| | *Oestrogen Receptor Values (fmol l⁻¹)* | | | | |
|---|---|---|---|---|---|
| | <30 | | 30+ | | |
| *Grade* | *No.* | *%* | *No.* | *%* | *Totals* |
| I | 32 | 42 | 44 | 58 | 76 |
| II | 59 | 45 | 73 | 55 | 132 |
| III | 30 | 63 | 18 | 37 | 48 |
| Totals | 121 | | 135 | | 256 |

**Figure 1** Comparison of histological grades.

**Table IV(A)** The 539 cases by treatment: comparison of observed and expected events, i.e. recurrences and deaths in each histological grade. (Stratified by menopausal and nodal status and treatment)

| *Grade* | *Number of patients* | *Observed events (O)* | *Expected events (E)* | *Ratio* |
|---|---|---|---|---|
| I 'Nolvadex' | 101 | 28 | 38.0 | 0.74 |
| No treatment | 87 | 40 | 30.0 | 1.33 |
| II 'Nolvadex' | 139 | 49 | 66.9 | 0.73 |
| No treatment | 134 | 72 | 54.1 | 1.33 |
| III 'Nolvadex' | 38 | 26 | 27.0 | 0.96 |
| No treatment | 40 | 28 | 27.0 | 1.03 |

**Table IV(B)** The 539 cases by treatment: comparison of observed and expected deaths in each histological grade. (Stratified by menopausal and nodal status and treatment)

| *Grade* | *Number* | *Observed deaths (O)* | *Expected deaths (E)* | *Ratio (O/E)* |
|---|---|---|---|---|
| I 'Nolvadex' | 101 | 22 | 26.3 | 0.84 |
| No treatment | 87 | 27 | 22.7 | 1.19 |
| II 'Nolvadex' | 139 | 39 | 51.6 | 0.76 |
| No treatment | 134 | 57 | 44.4 | 1.28 |
| III 'Nolvadex' | 38 | 25 | 25.2 | 0.99 |
| No treatment | 40 | 25 | 24.8 | 1.01 |

**Figure 2** Effect of 'Nolvadex' within histological grades.

reveal that the observed to expected ratios are less than one in each of the treated groups indicating a treatment benefit in each tumour grade, although the difference in ratios between the treated and untreated groups is less for grade III than for either grades I or II.

### Discussion

These results demonstrate very clearly that histological grading is predictive in terms of events and survival. These findings agree with results from other large studies concerning the prognosis of breast cancer (Wallgren *et al.*, 1984; Rose *et al.*, 1985). Histological grading is often criticised because of its subjective nature and observer variation. Furthermore, the histological specimen itself may not be truly representative of the tumour as a whole. Nevertheless, with one observer studying all 546 sections we are confident that the subsequent interpretations of the data are secure. The relatively high number of grade I tumours in the sample might reflect random bias of tissue samples available for analysis or the disproportionate number of node negative cases in the trial as a whole (NATO, 1983).

For the purpose of classifying oestradiol receptor status in this study, a cut-off point of 30 fmol $mg^{-1}$ cytosol protein was chosen as this represented a median value for the trial as a whole. Using lower values of cut-off to discriminate between positive and negative tumours does not materially affect the result, although it weakens statistical comparisons. At a 30 fmol $mg^{-1}$ cytosol protein cut-off point there is little difference between grades I and II but histological grade III tumours were almost twice as likely to be receptor negative than receptor positive, according to our definition. There was thus a clear statistically significant difference in ER content between grades I and II combined and grades III, adding further support to previous observations showing a correlation between histological grade and oestrogen receptor status (Elston *et al.*, 1980).

The results indicate that adjuvant tamoxifen may be of greatest benefit in the better differentiated (Grades I and II) tumours which are also the tumours likely to have a greater oestradiol receptor content. This result provides some support for the notion that the differences in outcome from adjuvant tamoxifen between different biological subsets of breast cancer is one of magnitude rather than kind. Paradoxically, the overview analysis of all trials of adjuvant tamoxifen (Peto, personal communication) has suggested that the relative risk reductions, based on lymph node status, are constant, thus the greater the risk of relapse the greater the absolute benefit of adjuvant tamoxifen.

Clearly, however, such detailed advice to clinicians must await analyses on histological grading from other adjuvant tamoxifen trials.

### References

BLOOM, H.J.G. & RICHARDSON, W.W. (1957). Histological grading and prognosis in breast cancer: A study of 1409 cases of which 395 have been followed for 15 years. *Br. J. Cancer*, **11**, 359.

BLOOM, H.J.G. (1962). The role of histological grading in the study of breast cancer. *Acta Un. Int. Cancer*, **18**, 843.

ELSTON, C.W., BLAMEY, R.W., JOHNSON, J., BISHOP, H.M., HAYBITTLE, J.K. & GRIFFITHS, K. (1980). The relationship of oestradiol receptor (ER) and histological tumour differentiation with prognosis in human primary breast carcinoma. In: *Breast cancer experimental and clinical aspects*. Mouridsen H. & Palshof T. (eds), p. 49. Pergamon press, New York.

McGUIRE, W.L., PEARSON, O.H. & SEGALOFF, A. (1975). Predicting hormone responsiveness in human breast cancer. In: *Estrogen Receptors in Human Breast Cancer*, McGuire *et al.* (eds), p. 17. Raven Press, New York.

NOLVADEX ADJUVANT TRIAL ORGANISATION (1983). Controlled trial of tamoxifen as adjuvant agent in management of early breast cancer. *Lancet*, **i**: 257.

NOLVADEX ADJUVANT TRIAL ORGANISATION (1985). Improved survival amongst patients treated with adjuvant tamoxifen after mastectomy for early breast cancer. *Lancet*, **ii**, 450.

NOLVADEX ADJUVANT TRIAL ORGANISATION (1988). Controlled trial of tamoxifen as a single adjuvant agent in the management of early breast cancer. *Br. J. Cancer*, **57**, 608.

PETO, R. (1987). Personal communication.

PETO, R. (1976). Design and analysis of randomised clinical trials requiring prolonged observation of each patient. I. Introduction and Design. *Br. J. Cancer*, **34**, 585.

PETO, R. (1977). Design and analysis of randomised clinical trials requiring prolonged observation of each patient. II. Analysis and Examples. *Br. J. Cancer*, **35**, 1.

ROSE, C., THORPE, S. ANDERSON, K.W. & 4 others (1985). Beneficial effects of adjuvant tamoxifen therapy in primary breast cancer patients with high oestrogen receptor values. *Lancet*, **i**, 16.

WALLGREN, A., GLAS, V., GRESTAFSSEN, S., SKOOQ, L., THEVE, N.O. & NORDENSKJOLD, B. (1984). Effects of adjuvant therapy in primary breast cancer in relation to the oestrogen receptor level. *Recent Results Cancer Res.* **91**, 215.